# Bioactive Compounds of *Sideritis* Species in Inflammation, Neuroprotection and Cardiometabolic Health: A Review

**DOI:** 10.3390/ijms27146217

**Published:** 2026-07-12

**Authors:** Gonxhe Kajtazi Çitaku, Joanna Harasym

**Affiliations:** 1 Department of Food Technology, Faculty of Food Technology, University ‘’Isa Boletini’ Str. Ukshin Kovacica, 40000 Mitrovica, Kosovo; gonxhe.kajtazi@umib.net; 2 Department of Biotechnology and Food Analysis, Wroclaw University of Economics and Business, Komandorska 118/120, 53-345 Wroclaw, Poland; 3 Adaptive Food Systems Accelerator-Science Centre, Wroclaw University of Economics and Business, 53-345 Wroclaw, Poland

**Keywords:** *Sideritis*, verbascoside, bioactive compounds, NF-κB/NLRP3 signaling, neuroprotection, oxidative stress, inflammation

## Abstract

*Sideritis* species (Lamiaceae), the aromatic mountain teas of the Mediterranean and Balkans, accumulate a phytochemically rich array of bioactive compounds—phenylethanoid glycosides (notably verbascoside), flavonoid aglycones and glycosides, phenolic acids, and terpenoids—whose pharmacological relevance is increasingly defined at the molecular level. This review synthesizes current evidence on the molecular mechanisms through which these constituents act in human health and disease, integrating in vitro, in vivo, in silico, and clinical data. Anti-inflammatory activity is attributed to modulation of the NF-κB and NLRP3 inflammasome pathways; direct verbascoside binding to both targets has been demonstrated in vitro by microscale thermophoresis (nanomolar-to-micromolar Kd), with suppression of TNF-α and IL-1β observed in cell-based and animal models. Neuroprotective effects are proposed to operate through amyloid-β clearance via ADAM10 upregulation and enhanced microglial phagocytosis in transgenic mouse models, together with in vitro triple monoamine (serotonin, noradrenaline, dopamine) reuptake inhibition, and are supported at the functional level by small randomized clinical trials reporting cognitive and anxiolytic benefits. Antioxidant and cytoprotective actions reflect radical scavenging, metal chelation, and restoration of SOD/CAT/GSH with attenuation of lipid peroxidation, underpinning documented hepatoprotective and gastroprotective outcomes. Preliminary cardiometabolic findings from small clinical trials include cholesterol modulation and sex-dependent effects on insulin sensitivity that remain to be confirmed in larger, adequately powered cohorts. We further evaluate structure–activity relationships, species- and tissue-level chemical variability, and green extraction strategies relevant to standardized bioactivity-preserving preparations. Critical gaps—in vivo validation of antiproliferative effects documented only in cell-based and in silico studies so far, systematic toxicological assessment, and bioavailability characterization—are identified to guide translation. Collectively, *Sideritis*-derived phytochemicals, exemplified by *S. scardica*, represent a mechanistically coherent group of plant bioactives with defined molecular targets in inflammatory, neurodegenerative, and metabolic disorders.

## 1. Introduction

**Figure 1:**
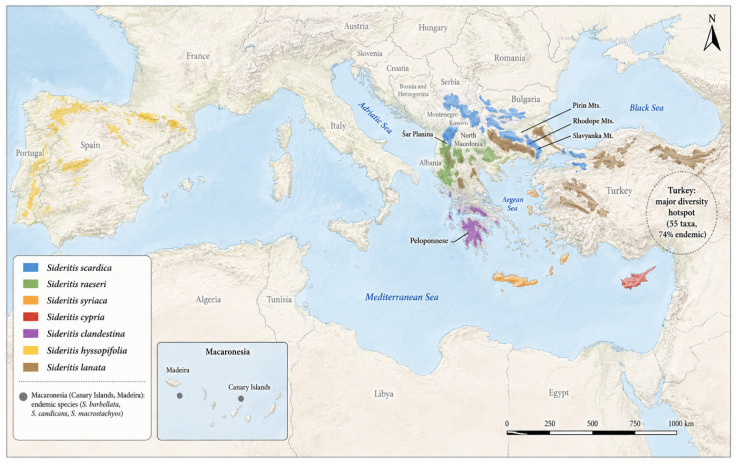
Geographical distribution of principal *Sideritis* species across the Mediterranean basin and the Balkan peninsula [1,3].

The genus *Sideritis* L. (Lamiaceae) comprises over 150 annual and perennial aromatic species whose name derives from the Greek “σίδηρος” (iron), reflecting their ancient use as wound-healing poultices for injuries from iron weapons [1,2]. Distributed across the Mediterranean basin, the Balkan Peninsula, Macaronesia, and Western Asia, these plants occupy mountainous, xerophytic habitats typically above 800 m [1,3]. The genus is markedly endemic—Türkiye alone hosts 55 taxa, 74% of them endemic—while the southern Balkans form a secondary center of diversity for *S. scardica* and *S. raeseri* (Figure 1) [2,4]. Intense hybridization among sympatric populations makes *Sideritis* one of the most taxonomically challenging genera in the Lamiaceae and underlines the importance of biodiversity conservation [2,4].

Known colloquially as “mountain tea,” “ironwort,” or “shepherd’s tea,” *Sideritis* species have been consumed as herbal infusions across the Mediterranean and Balkans for centuries; traditional preparations of the aerial parts are used to relieve respiratory disorders, gastrointestinal discomfort, fever, pain, and anxiety [1,2]. This therapeutic reputation gained formal recognition when the European Medicines Agency (EMA) listed *S. scardica*, *S. clandestina*, S. *raeseri*, and *S. syriaca* as traditional herbal medicinal products for the symptomatic relief of the common cold and mild gastrointestinal complaints [5,6].

The pharmacological versatility of the genus rests on a chemically diverse pool of secondary metabolites: flavonoids (notably apigenin, luteolin, and isoscutellarein glycosides), phenylethanoid glycosides (verbascoside, lavandulifolioside), phenolic acids (chlorogenic, rosmarinic, and caffeic), diterpenes, iridoids, and volatile terpenoids [1,2,5]. This richness varies considerably with species, genotype, altitude, climate, and post-harvest processing, offering both opportunities for targeted bioactivity and challenges for standardization [3,6].

Scientific interest in *Sideritis* has intensified sharply over the past decade, with a recent bibliometric analysis documenting a marked rise in publications extending well beyond phytochemistry into pharmacology and clinical research [3]. Earlier reviews have addressed the genus’s uses, chemistry, and pharmacology [1,2], its essential-oil profiles [7], and individual species such as *S. raeseri* [8] and *S. cypria* [6]. What remains dispersed across primary studies, however, is the molecular-level evidence—the signaling pathways, protein targets, and mechanistic readouts through which *Sideritis* constituents exert their effects—that has emerged in recent years from in vitro, in vivo, in silico, and clinical work.

The present review consolidates this mechanistic evidence, with particular emphasis on S. *scardica*, at the interface of phytochemistry and human health and disease. Specifically, we: (1) survey the principal classes of *Sideritis* bioactive compounds and the structural features that govern their molecular activity; (2) synthesize mechanistic evidence across the genus’s best-characterized activities—antioxidant and cytoprotective signaling, anti-inflammatory modulation of the NF-κB and NLRP3 inflammasome pathways, neuroprotection via amyloid-β processing and monoamine-transporter inhibition, cardiometabolic and enzyme-level regulation, and antiproliferative signaling; (3) integrate in vitro, in vivo, in silico, and clinical findings around defined molecular targets; and (4) identify the principal gaps in mechanistic validation, safety assessment, and phytochemical standardization that must be addressed for therapeutic translation.

This review is based on a structured, non-systematic search of PubMed, Scopus, Web of Science, and Google Scholar, covering the literature from database inception through early 2026. Search strings combined the genus term “*Sideritis*” with phytochemical descriptors (e.g., “verbascoside,” “phenylethanoid glycoside,” “flavonoid,” “phenolic acid,” “essential oil,” “diterpene”) and bioactivity descriptors (e.g., “antioxidant,” “anti-inflammatory,” “NF-κB,” “NLRP3,” “neuroprotective,” “amyloid,” “antimicrobial,” “antiproliferative,” “cardiometabolic”). Reference lists of retrieved articles and earlier reviews were screened manually to identify additional primary sources. Peer-reviewed studies reporting the phytochemical characterization or biological activity of taxonomically identified *Sideritis* species were eligible for inclusion; reports lacking species-level identification, conference abstracts without accessible full data, and studies not concerning the genus were excluded, with non-English records considered only where an English abstract permitted appraisal. Evidence was weighted hierarchically when integrating mechanistic claims: randomized controlled and other human clinical trials were given the greatest weight for translational relevance, followed by in vivo animal models, with in vitro and in silico data (including molecular docking and microscale thermophoresis) treated as mechanistic and hypothesis-generating evidence requiring experimental confirmation. Priority was given throughout to findings related to a defined, chemically characterized extract or marker compound.

## 2. Phytochemical Composition and Bioactive Compounds

### 2.1. Primary Bioactive Constituents

**Figure 2:**
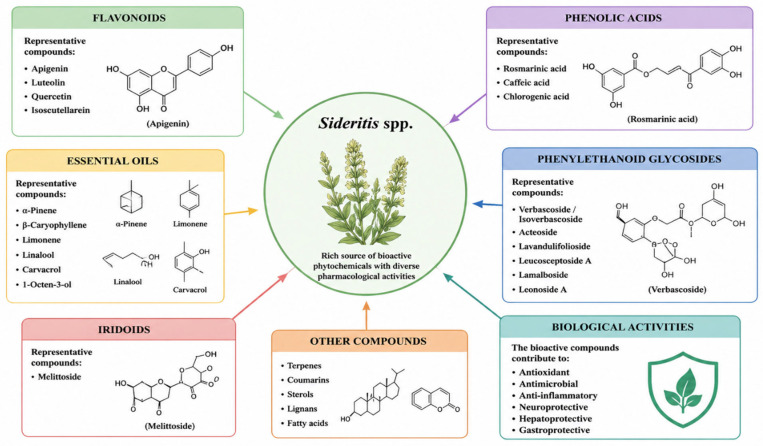
Schematic overview of the major classes of phytochemicals identified in *Sideritis* species [1,2].

*Sideritis* species accumulate a rich and diverse set of bioactive compounds—terpenes, flavonoids, phenylethanoid glycosides, essential oils, iridoids, coumarins, sterols, and lignans—that together account for the pharmacological activity of the genus (Figure 2). The structural diversity within each class is the molecular basis of the multi-target activity profile discussed in Section 3.

#### 2.1.1. Flavonoids

Flavonoids are the most extensively characterized class in *Sideritis*, occurring in both aglycone and glycosidic forms. LC-MS/MS-QTOF analysis of *S. raeseri*, *S. scardica*, and *S. syriaca* across nine Greek populations identified 17 flavonoids among 23 total secondary metabolites (87% of identified compounds), with four detected consistently: verbascoside/isoverbascoside, apigenin 7-*O*-glucoside, isoscutellarein 7-*O*-[6″-*O*-acetyl]-allosyl(1 → 2)-glucoside, and apigenin 7-(4″-*p*-coumaroylglucoside) [5]. The main aglycones—isoscutellarein (8-hydroxyapigenin), hypolaetin (8-hydroxyluteolin), and apigenin—together with their methylated and acetylated glycosidic derivatives, form a substitution pattern characteristic of *Sideritis* section *Empedoclia* [2,5].

Glycosylation patterns are pharmacologically significant: glycosides such as apigenin 7-*O*-glucoside show enhanced water solubility and intestinal absorption, while acylated forms such as apigenin 7-(4″-*p*-coumaroylglucoside) may exhibit prolonged activity owing to delayed gastrointestinal hydrolysis [2]. Dendrogram analysis showed that *S. syriaca* (Crete) and *S. scardica* (Peloponnese, Kastoria) cluster together while *S. raeseri* (Kastoria) is chemically distinct, indicating that species-level genetics outweigh geography in flavonoid distribution [5].

In cultivated *S. cypria*, four isoscutellarein and hypolaetin derivatives were identified in flower infusions, with flowers containing markedly higher total flavonoid content (32.00 mg rutin equivalents/g DW) than leaves (7.72 mg/g DW) and stronger antioxidant activity (DPPH IC_50_ = 267.9 vs. 793.0 µg/mL) [6], identifying flowers as the richer source of antioxidant-active flavonoids. LC-MS/MS profiling of *S. pisidica* revealed luteolin-7-glucoside, apigenin 7-glucoside, hesperidin, and kaempferol alongside phenolic acids [9], while LC-ESI-MS/MS quantification of *S. perfoliata* showed apigenin 7-glucoside at 1437.5 µg/g in water extracts and total flavonoids of 40.90 mg quercetin equivalents/g in methanol extracts [10].

#### 2.1.2. Phenylethanoid Glycosides

Phenylethanoid glycosides are the second major non-volatile class. Four were identified across *S. raeseri*, *S. scardica*, and *S. syriaca*, including verbascoside (acteoside) and isoverbascoside [5]. *S. cypria* accumulates a broader range—five glycosides in flower infusions (acteoside, leucosceptoside A, lamalboside, leonoside A) plus lavandulifolioside in leaves, with structures confirmed by high-field NMR [6].

*S. perfoliata* provides the most detailed verbascoside quantification: 50,951.1 µg/g DW in water extracts, 29,033.8 µg/g in methanol, and only 136.8 µg/g in ethyl acetate, representing 65.3% and 73.4% of total phenolics in water and methanol extracts, respectively, establishing it as the dominant secondary metabolite [10]. Mechanistically, verbascoside’s caffeoyl catechol ring supports radical scavenging through hydrogen-atom and single-electron transfer, while its sugar residues modulate hydrophilicity and tissue distribution [1]; these properties underlie the anti-inflammatory, antioxidant, and cytoprotective activity discussed in Section 3 [11].

#### 2.1.3. Phenolic Acids

Chlorogenic, rosmarinic, and caffeic acids are the principal phenolic acids reported. Chlorogenic acid occurs consistently in Balkan and Cypriot species [5,6] and is the second most abundant compound in *S. perfoliata*: 24,933.4 µg/g (water) and 9975.8 µg/g (methanol), with documented antioxidant, anti-inflammatory, anti-diabetic, anti-obesity, and anti-hypertensive effects [5,10]. Rosmarinic acid, a caffeic acid ester of 3,4-dihydroxyphenyllactic acid, exhibits stronger DPPH/ABTS scavenging than caffeic acid owing to two catechol moieties providing four phenolic hydroxyls [2]. It dominates the phenolic profile of *S. pisidica* [9] but in *S. perfoliata* was detected only in ethyl acetate extracts (1.52 µg/g), illustrating solvent-dependent recovery [10].

#### 2.1.4. Essential Oils and Terpenoids

The volatile fraction comprises monoterpenes, sesquiterpenes, alcohols, and aldehydes. GC-MS analysis of cultivated *S. cypria* revealed organ-specific profiles: flower oils were dominated by monoterpene hydrocarbons (88.4%)—α-pinene (38.0%), β-phellandrene (25.8%), β-pinene (14.7%)—while leaf oils contained higher sesquiterpene hydrocarbons (23.4%) and oxygenated sesquiterpenes (22.2%), with β-caryophyllene (22.5%) and caryophyllene oxide (8.3%) as major constituents; yields differed correspondingly (0.25% flowers vs. 0.03% leaves) [6]. Substantial interspecific variability in volatile profiles is documented across the genus [7]. Of particular molecular interest, β-caryophyllene is a selective CB2 receptor agonist (and FDA-approved food additive) that contributes anti-inflammatory and neuroprotective activity through cannabinoid-receptor signaling [2].

#### 2.1.5. Diterpenes, Iridoids, and Minor Constituents

*Sideritis* species also accumulate *ent*-kaurene-type diterpenes and iridoid glycosides. The iridoid melittoside was identified in *S. cypria* leaf and flower infusions alongside chlorogenic acid [6]. Cultivated *S. cypria* was notably iridoid-poor compared with *S. perfoliata* subsp. *perfoliata* from the same region, which accumulated six iridoid glycosides, indicating species-level variation within section *Empedoclia* [6]. Lignans (e.g., pinoresinol at 12.18 µg/g in *S. perfoliata* water extracts [10]), coumarins, and phytosterols are reported but remain poorly characterized [2,4].

#### 2.1.6. Environmental and Genetic Variability

Phytochemical synthesis is modulated by genetic variation, altitude, temperature, UV exposure, soil composition, agricultural practices, and harvest timing [2,3]. Wild-harvested *S. cypria* from mountain habitats (500–750 m) differed from cultivated lowland plants in essential-oil profile and yield, though non-volatile metabolites remained comparable, supporting cultivation without loss of the bioactive constituents [6]. Within *S. raeseri*, populations from Othrys, Kastoria, and Elassona shared only 10 phenolic compounds, highlighting the need for population-specific quality control [5].

Across the genus, *S. scardica* is the most phytochemically documented taxon, with the broadest evidence for flavonoid and phenolic-acid richness [1,5]. *S. cypria* is distinguished by its diverse phenylethanoid glycoside profile (5–7 glycosides, including the rare trisaccharide lamalboside) and higher bioactive content in flowers than leaves [6]. *S. perfoliata* shows the highest reported verbascoside concentration in the genus (50,951 µg/g, water extract) and the first reports of protocatechuic, 4-hydroxybenzoic, vanillic, and caffeic acids [10]. *S. pisidica* features a rosmarinic acid-dominated profile with anti-inflammatory effects on melanoma cells [9], and *S. congesta* has been investigated for cytoprotective potential supporting hepatoprotection in acetaminophen-induced toxicity models [12].

The major bioactive compounds, with compound class, key structural features, quantitative concentrations (where available), species of occurrence, analytical method, and references, are summarized in Table 1.

### 2.2. Recovery of Bioactive Constituents and Solvent-Dependent Profiles

Because reported bioactivities depend directly on which constituents an extract contains, extraction conditions are a key determinant of the molecular profile available for pharmacological study. Solvent polarity, technique, temperature, duration, and solid-to-solvent ratio jointly govern recovery while affecting thermolabile compounds [13].

The clearest demonstration in the genus comes from *S. perfoliata* [10]: water recovered the highest total phenolics (52.18 mg GAE/g) and verbascoside (50,951 µg/g), methanol gave the highest flavonoid content (40.90 mg QE/g) and the most complete profile (20 vs. 14 vs. 9 compounds across water, methanol, and ethyl acetate), and rosmarinic acid appeared only in ethyl acetate while hesperidin, (-)-epicatechin, and 2,5-dihydroxybenzoic acid appeared only in methanol. No single solvent captures the full phytochemical complexity—an important caveat, since mechanistic studies built on a single extract may under-sample the active constituents. Deveci et al. (2019) confirmed distinct phenolic and flavonoid profiles between methanol and water extracts of other *Sideritis* species, and Erdoğmuş et al. (2025) recovered a rosmarinic acid-dominated profile from *S. pisidica* using ethanol, further illustrating solvent-driven chemical signatures [9,14].

Among greener approaches, natural deep eutectic solvent (NADES) extraction offers high recovery with low-toxicity, GRAS-status components. Karadendrou et al. (2025) identified a betaine-1,3-propanediol system for *S. scardica* yielding 49.2 mg GAE/g total phenolics and 45.9 mg CAE/g total flavonoids—roughly double a matched 70% ethanol extract—with the practical advantage that GRAS components avoid residual-solvent concerns for downstream biological testing [15]. Ultrasound-, microwave-, and pressurized-liquid-assisted techniques similarly enhance recovery while preserving heat-sensitive flavone glycosides [14,16]. For mechanistic and clinical work, the operative priority is standardized quantification of marker compounds—verbascoside, apigenin 7-O-glucoside, chlorogenic acid, and rosmarinic acid—so that biological activity can be related to a defined chemical input and compared across studies [6,10].

## 3. Biological Activities and Pharmacological Potential

Bioactive capacity of compounds in *Sideritis* genus is broad and multifunctional (Table 2).

### 3.1. Antioxidant Activity and Redox Signaling

The antioxidant capacity of *Sideritis* is among the most extensively documented properties of the genus and derives primarily from its phenolic composition—flavonoids and hydroxycinnamic-acid derivatives—which neutralize reactive oxygen species through radical scavenging, metal chelation, and inhibition of lipid peroxidation [1,2] Table 2.

Quantitative profiling of *S. perfoliata* revealed strong solvent-dependent variation: water extracts showed the highest radical scavenging (DPPH 405.53 mg TE/g; ABTS 149.71 mg TE/g) and reducing power (CUPRAC 210.69 mg TE/g; FRAP 161.19 mg TE/g), followed by methanol (DPPH 266.25 mg TE/g), with ethyl acetate weakest across all assays [10]. These differences correlated strongly with total phenolic content (36.68–52.18 mg GAE/g) and verbascoside concentration (50,951 vs. 136.8 µg/g in water vs. ethyl acetate) [10].

### 3.2. Anti-Inflammatory and Immunomodulatory Effects

The anti-inflammatory action of *Sideritis* extends beyond antioxidant protection to direct modulation of inflammatory signaling and cytokine networks, documented in in vivo models, cell-based assays, and molecular docking studies [1,2].

The most thorough in vivo characterization used carrageenan-induced rat paw edema with *S. scardica* extracts: diethyl ether and n-butanol fractions produced ~50% inflammation reduction at 200 and 100 mg/kg, respectively, comparable to indomethacin (50% at 4 mg/kg), with HPLC fingerprinting attributing the effect primarily to apigenin, luteolin, and their glycosides; all four fractions also showed dose-dependent gastroprotection comparable to ranitidine against ethanol-induced gastric lesions [18]. At the cellular level, *S. pisidica* extracts modulated pro-inflammatory cytokine production in melanoma cells, increasing TNF-α, TGF-β, DEF-β2, and IL-1β at IC_50_ concentrations—an immunostimulatory rather than immunosuppressive profile in the tumor microenvironment, suggesting these extracts may activate anti-tumor immune responses while providing antioxidant cytoprotection to normal tissues [9].

Complementary evidence from *S. bilgeriana* showed that its methanolic extract reduced cell migration and attenuated TNF-α and IL-1β in a pleurisy model, with effects correlated to NF-κB suppression; antinociceptive activity in neuropathic pain models was likewise associated with decreased TNF-α and NF-κB expression [19]. *S. congesta* extracts showed cytoprotective potential in acetaminophen (APAP)-induced hepatotoxicity in HepG2 cells, restoring SOD and CAT activity, maintaining GSH, mitigating MDA elevation, and demonstrating anti-mutagenic activity [12].

The molecular basis of these effects has been examined computationally and biophysically. In silico virtual screening of 657 *Sideritis* metabolites identified verbascoside and apigenin 7,4′-bis(trans-p-coumarate) as the most potent predicted binders of both the NLRP3 inflammasome and NF-κB; this prediction was tested directly in vitro by microscale thermophoresis, which confirmed verbascoside binding with K_d values of 0.67 ± 0.18 µM (NLRP3) and K_d ≈ 0.01 µM (NF-κB) [20]. The docking prediction together with the direct binding measurement provides a molecular rationale for the anti-inflammatory activity of verbascoside-rich preparations, although suppression of downstream pathway output in a cellular NF-κB/NLRP3 readout was not measured in that study and remains to be confirmed [20]. Because NF-κB and NLRP3 signaling sit at the intersection of chronic inflammation and tumorigenesis, the same binding profile has been proposed to underline both the anti-inflammatory and the anti-cancer activity of verbascoside—a link examined further in Section 4.4 [20].

### 3.3. Antimicrobial and Antifungal Properties

*Sideritis* essential oils and extracts exhibit broad-spectrum activity against Gram-positive and Gram-negative bacteria and pathogenic fungi, acting principally through disruption of microbial membranes and synergistic terpenoid–phenolic interactions [7].

The *S. syriaca* subsp. syriaca oil, rich in carvacrol (33.68%), showed the strongest antibacterial activity, with MIC values of 0.60–2.25 mg/mL against Gram-positive (*Staphylococcus aureus*, *S. epidermidis*) and Gram-negative organisms (*Pseudomonas aeruginosa*, *Enterobacter cloacae*, *Klebsiella pneumoniae*, *Escherichia coli*)—significantly stronger than carvacrol or α-/β-pinene alone, indicating synergistic component interactions [7]. The *S. euboea* oil was particularly potent against Gram-positive organisms (MIC 3.25–6.51 µg/mL against *Micrococcus luteus*, *Enterococcus faecalis*, *S. aureus*, *S. epidermidis*), approaching amikacin and ampicillin efficacy [7].

*Sideritis* oils typically show greater Gram-positive than Gram-negative activity, with *S. aureus* most consistently susceptible and *P. aeruginosa* generally resistant; the *S. italica* oil was an exception, showing higher activity against *P. aeruginosa* (MIC 3.9–7.8 µg/mL) than *S. aureus* (62.5–125.0 µg/mL) and additional activity against *Helicobacter pylori* [7]. For antifungal activity, the *S. bilgeriana* and *S. cilicica* oils—both rich in β-pinene (39.1% and 48.4%)—showed MIC values of 0.03 mg/mL against *Candida albicans*, matching ketoconazole, while the *S. cypria* oil produced a 24.67 mm inhibition zone against *C. albicans* at 10 µL, exceeding nystatin (20.33 mm) [7]. Polar extracts of *S. scardica* from different origins also showed antimicrobial activity, particularly against Gram-positive pathogens and *Candida* spp., with Türkiye-origin material the strongest [17]. These data support the traditional anti-infective use of the genus and identify carvacrol- and pinene-rich oils as the most active chemotypes.

### 3.4. Neuroprotective and Cognitive Enhancement

The neuroprotective properties of *Sideritis* are perhaps the most distinctive pharmacological feature of the genus, supported by in vitro, in vivo, and human clinical evidence and operating through three complementary molecular mechanisms: amyloid-β clearance and plaque reduction, monoamine-transporter inhibition, and cerebral blood-flow modulation.

The most compelling pre-clinical data come from an animal model—APP-transgenic mice, with vehicle-treated transgenic and non-transgenic littermates as controls. Daily oral *S. scardica* and *S. euboea* extracts (individually and combined) produced dose-dependent reductions in intracerebral soluble Aβ42: post-onset treatment decreased Aβ42 by 58% (*S. scardica*), 60% (*S. euboea*), and 56% (combination) relative to vehicle-treated controls, and the combination reduced plaque numbers by 42% and plaque size by 25% [21]. Treatment rescued neuronal loss to non-transgenic levels, with combination therapy increasing cortical neuronal area by 50%; Morris water-maze escape latencies fell 55–61% on consecutive test days, and cognitive enhancement in non-transgenic aged mice persisted across longitudinal testing at 150, 300, 450, and 600 days (latency reductions 54–66%) [21]. These outcomes are reported as percentage changes relative to vehicle-treated controls with statistical significance (mean + SEM, *p* ≤ 0.05); the source does not report per-group confidence intervals or effect sizes for these reductions [22]. Mechanistically, these effects were accompanied by increased α-secretase ADAM10 expression and enhanced microglial phagocytic activity around plaques, indicating both reduced Aβ production and enhanced clearance [21].

A distinct mechanism was defined in vitro by characterizing *S. scardica* methanol extract as a triple monoamine reuptake inhibitor. It inhibited serotonin, noradrenaline, and dopamine uptake into rat brain synaptosomes with EC_50_ values of 31.0 µg/mL (95% CI 16.4–58.6), 42.3 µg/mL (95% CI 31.8–56.4), and 37.0 µg/mL (95% CI 27.5–49.8), respectively, with maximal inhibition of 108 ± 6%; in human JAR cells expressing hSERT the extract was markedly more potent (EC_50_ 1.4 µg/mL), and co-incubation with fluvoxamine produced a leftward shift in the dose–response curve (EC_50_ reduced from 3.8 to 0.5 nM with 50 µg/mL extract) [22]. This triple-transporter profile parallels pharmacological targets in depression, ADHD, and cognitive decline at the mechanistic level; whether it accounts for the clinical effects below has not been established directly.

These mechanisms are accompanied by measurable effects on human cognition in a single moderately sized trial. A double-blind, randomized, placebo-controlled, parallel-groups trial randomized 155 healthy adults aged 50–70 to daily 475 mg or 950 mg *S. scardica* extract, 240 mg *Ginkgo biloba* (active control), or placebo for 28 days; outcome-specific analyzed subsamples were smaller (cognition *n* = 140, mood *n* = 142, blood pressure *n* = 133, cerebral blood flow *n* = 57). Relative to placebo, the 950 mg dose significantly reduced false alarms on the Rapid Visual Information Processing task (post hoc *p* = 0.017, Cohen’s d = 0.66) and significantly decreased state anxiety relative to both placebo and the active *Ginkgo biloba* control [23]. Near-infrared spectroscopy in a subsample (*n* = 57) showed both doses significantly increased prefrontal-cortex oxygenated hemoglobin and oxygen saturation during cognitively demanding tasks on Day 1—acute cerebral blood-flow modulation. These hemodynamic effects were not sustained at Day 28, suggesting chronic cognitive improvement operates through monoaminergic mechanisms (specifically anxiety reduction) rather than sustained cerebral blood-flow changes [23].

Additional molecular relevance comes from in vitro cholinesterase inhibition (single-concentration enzymatic assays): the *S. albiflora* oil showed 22.1% AChE inhibition and a BChE IC_50_ of 157.2 µg/mL, while the *S. pisidica* oil produced 58.37% BChE inhibition at 200 µg/mL relative to galantamine (82.23%) [7,14]. The *S. perfoliata* water extract showed AChE and BChE inhibition alongside α-glucosidase and α-amylase inhibitory activity, suggesting dual neuroprotective and anti-diabetic potential through overlapping phenolic constituents [10].

## 4. Disease-Relevant Mechanisms and Therapeutic Potential

**Figure 3:**
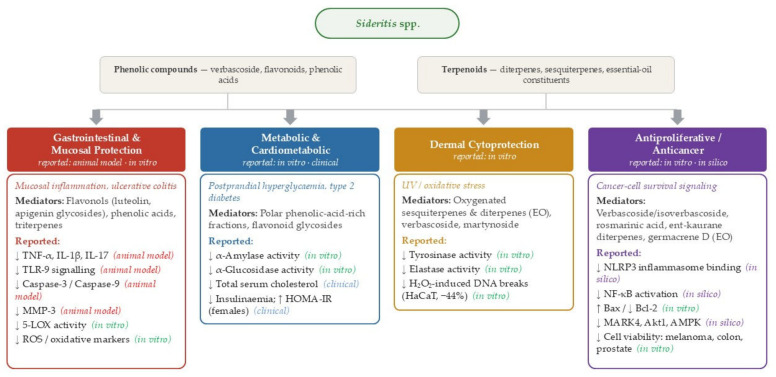
Reported molecular readouts associated with *Sideritis* bioactivity, grouped by disease context. Evidence type is indicated for each readout (in vitro, *animal model*, *clinical*, in silico). ↑—increase; ↓—decrease.

*Sideritis* species have a long ethnopharmacological history as remedies for inflammation, gastrointestinal complaints, respiratory infections, and nervous-system disorders [2]. Pre-clinical and clinical research over the past decade have begun to map these uses onto defined molecular mechanisms across four disease-relevant domains: gastrointestinal inflammation and mucosal protection, metabolic and cardiometabolic regulation, dermal cytoprotection, and antiproliferative/anticancer signaling (Figure 3).

### 4.1. Gastrointestinal Inflammation and Mucosal Protection

Traditional use of mountain tea for stomach ailments, ulcers, and intestinal disturbances is among the oldest ethnobotanical records for the genus, and the EMA monograph formally recognizes infusions of *S. scardica*, *S. clandestina*, *S. raeseri*, and *S. syriaca* as traditional herbal medicines for colds, coughs, and mild gastrointestinal disorders [2]. Experimental work has progressively moved from classical ulcer models toward mechanistic studies of inflammatory bowel disease.

Early in vivo work showed that decoctions of four Spanish *Sideritis* species reduced indomethacin- and stress-induced gastric ulceration in rats, with activity comparable to ranitidine [26]. The gastroprotective flavonoid hypolaetin-8-O-β-D-glucoside, found across several species including *S. leucantha* and *S. mugronensis*, reduces gastric lesions by increasing mucus production and decreasing gastric acid output [2]. At the cellular level, *S. scardica* n-butanol and diethyl ether extracts produced dose-dependent gastroprotection in an ethanol-induced acute gastric-damage model, with concomitant anti-inflammatory activity in the carrageenan paw-edema assay attributed to flavonol and phenolic-acid content [19].

The strongest mechanistic evidence concerns ulcerative colitis. In an acetic acid-induced rat colitis model, *S. perfoliata* ethanolic extract at 200 mg/kg significantly reduced colon-tissue TNF-α, IL-1β, and IL-17 to levels comparable to sulfasalazine; it also suppressed TLR-9 signaling, reduced MMP-3 activity, and attenuated the mitochondrial apoptotic markers caspase-3 and caspase-9 [25]. Oxidative stress (luminol and lucigenin chemiluminescence) was reduced in parallel, consistent with strong in vitro DPPH/ABTS scavenging (IC_50_ ~100.5 and ~90.5 µg/mL) and potent 5-lipoxygenase inhibition (IC_50_ 17.6 µg/mL, exceeding indomethacin at 21.4 µg/mL); histology confirmed reduced mucosal and glandular damage and decreased submucosal edema. The authors attributed these effects to synergy among the extract’s high flavonoid (407 mg QE/g), phenolic-acid (141 mg GAE/g), and triterpene (86 mg oleanolic-acid equivalent/g) contents—the first mechanistic validation of *S. perfoliata* for colitis specifically [25].

### 4.2. Metabolic and Cardiometabolic Regulation

*Sideritis* extracts inhibit key carbohydrate-metabolizing enzymes, directly relevant to traditional anti-diabetic use. The ethyl acetate fraction of *S. perfoliata* showed the strongest α-glucosidase and α-amylase inhibition, with IC_50_ values lower than acarbose for certain fractions, linked by HPLC to high phenolic-acid and flavonoid content [10]. *S. bilgeriana* extracts likewise showed concentration-dependent inhibition of both enzymes, with the most polar fractions most active [19]. Mechanistically, α-amylase inhibition reduces postprandial starch breakdown while α-glucosidase inhibition delays intestinal glucose absorption—the dual mechanism underlying acarbose’s action in type 2 diabetes. Essential oils contribute additionally: the *S. galatica* oil inhibited α-glucosidase with an IC_50_ of 0.632 mg/mL, outperforming acarbose (2.062 mg/mL), tentatively linked to its monoterpene hydrocarbons [7].

These enzyme-level effects (in vitro) are paralleled by cardiometabolic endpoints reported in a small human trial. In a single one-month placebo-controlled study, *S. euboea* extract significantly lowered total serum cholesterol in healthy adults, with no adverse effects on liver enzymes or other cardiometabolic markers [27]. A sex-stratified analysis within the same trial reported reductions in insulinemia and improvements in HOMA-IR in females only, an effect the authors hypothesized to involve adiponectin signaling [27]. These are preliminary observations from a single small cohort: they suggest possible clinically relevant cardiometabolic activity (cholesterol homeostasis, insulin sensitivity) acting independently of global antioxidant pathways, but require confirmation in larger, adequately powered, sex-balanced trials before any therapeutic claim can be made [27].

More detailed cardiometabolic endpoints come from the standardized SidTea+™ preparation of *S. scardica* in a randomized, double-blind, placebo-controlled, parallel-design trial (1500 mg/day, 4 weeks; *n* = 28 completers, 14 per group, from 30 randomized; powered at 80% for a medium effect; placebo = maltodextrin) [24]. The extract produced within-group reductions in systolic blood pressure (−10.8 mmHg; *p* = 0.002; Cohen’s d = 0.84, 95% CI 0.07–1.61), mean arterial pressure (−4.5 mmHg; *p* = 0.026; d = 0.53, 95% CI −0.23–1.28), and resting heart rate (−3.1 bpm; *p* = 0.036; d = 0.26, 95% CI −0.48–1.01), alongside increased estimated VO_2_max (+1.1 mL/kg/min; *p* = 0.031) and improved total antioxidant capacity (*p* < 0.001) with reduced plasma lipid peroxidation [24]. These within-group changes reached statistical significance, but the corresponding effect-size 95% confidence intervals crossed zero for mean arterial pressure, resting heart rate, and VO_2_max; only systolic blood pressure (within-group) and the between-group reduction in lipid peroxidation (TBARS; d = −0.90, 95% CI −1.68 to −0.12) yielded effect-size intervals excluding zero, indicating that several effects should be read as preliminary given the small cohort [24]. The liver-enzyme and herb–drug-interaction safety aspects of this and related trials are considered in Section 5.

### 4.3. Dermal Cytoprotection and Oxidative-Stress Defense

Beyond systemic activity, *Sideritis* constituents act on enzyme targets and oxidative-stress pathways relevant to skin biology. The first comprehensive study across multiple polarity fractions of the endemic *S. sipylea* found that all extracts inhibited tyrosinase at 150 µg/mL (maximum ~26% for the methanol and water/methanol fractions), with inhibition correlating significantly with total phenolic content and DPPH scavenging—implicating verbascoside, martynoside, and acetylated isoscutellarein glycosides as the active compounds [28]. For elastase, which cleaves dermal elastin, the *S. sipylea* oil showed the highest anti-elastase activity (12.84% at 0.5 µg/mL), attributed to oxygenated sesquiterpenes and diterpenes; comparable elastase inhibition was reported for *S. perfoliata* ethanol extract [29] and anti-tyrosinase activity for *S. albiflora* and *S. leptoclada* oils, inferior to kojic acid but appreciable [14].

The most molecularly informative finding concerns cellular cytoprotection. Despite weak direct radical scavenging (DPPH IC_50_ 24.8 mg/mL; ABTS IC_50_ 1.27 mg/mL), *S. raeseri* subsp. *raeseri* oil reduced H_2_O_2_-induced DNA strand breaks in HaCaT keratinocytes by 44% at 0.5 mg/mL—protection consistent with activation of endogenous defensive pathways rather than direct quenching [30]. The same oil showed antiproliferative activity against melanoma (A375), colon adenocarcinoma (Caco2), and prostate cancer (PC3, DU145) lines (EC_50_ 0.114–0.216 mg/mL), bridging to the anticancer mechanisms below [30].

### 4.4. Antiproliferative and Anticancer Activity

Several *Sideritis* species show antiproliferative activity in vitro through diverse, increasingly well-defined molecular routes—a domain of particular relevance to the role of plant bioactives in disease. In cell-based assays, *S. pisidica* extract (rosmarinic acid-dominant) yielded an IC_50_ of 1.25 mg/mL against G361 human melanoma cells, and molecular docking predicted that rosmarinic acid, hesperidin, apigenin-7-glucoside, and caffeic acid bind with high affinity to the cancer-relevant targets MARK4, Bax, Akt1, and AMPK, respectively [9]. *S. leptoclada* ethanol extract induced apoptosis in HT144 melanoma cells by elevating TNF-α and triggering ROS-dependent cell death, while S. ozturkii extracts upregulated the pro-apoptotic proteins Bax and APAF in DLD-1 colon-cancer cells [9].

At the single-compound level, verbascoside was profiled against 49 NCI tumor cell lines, showing a distinctive resistance pattern—it partially overcame P-glycoprotein-mediated multidrug resistance with a resistance degree of only ~3-fold (vs. ~400-fold for doxorubicin), and its cytotoxicity correlated with specific proteomic signatures rather than classical drug-resistance proteins [20]. In the same study, in silico docking indicated verbascoside binding to NLRP3 and NF-κB (binding energies −14.3 and −10.4 kcal/mol), a shared mechanism that plausibly links the anti-inflammatory data of Section 3.2 to the antiproliferative findings here, though the connection remains computational [20].

Diterpenes contribute independently in cell-based assays. The *S. montana* oil (germacrene D 24.6%) produced IC_50_ values of 31–35 µg/mL against melanoma, breast, and colon carcinoma lines, while the *S. perfoliata* oil was active against amelanotic melanoma (C32) and renal adenocarcinoma (ACHN) at IC_50_ ~100 µg/mL, with linalool and trans-caryophyllene identified as the responsible components [7].

Across the genus, all antiproliferative evidence to date derives from cell-based cytotoxicity assays and computational docking; no in vivo tumor-bearing model or clinical study of any *Sideritis* preparation has been reported. These results should therefore be regarded as preliminary and hypothesis-generating, and require in vivo validation before any anticancer claim can be made.

## 5. Safety, and Translational Gaps

The efficacy trials of standardized *S. scardica* and *S. euboea* are presented alongside their mechanistic counterparts in Section 3.4 and Section 4.2; translating those findings into validated human benefit additionally requires dedicated safety characterization and pharmacokinetic understanding—areas supported by only a small body of work, almost entirely on standardized *S. scardica*.

The available clinical trials indicate a favorable tolerability profile. In the SidTea+™ trial (1500 mg/day, 4 weeks; *n* = 28 completers), liver-function markers moved in a favorable direction, with reductions in γ-GT (−3.7 U/L; *p* = 0.039) and SGPT (−3.3 U/L; *p* = 0.049) and no adverse biochemical signal [24]; likewise, *S. euboea* extract lowered total serum cholesterol without adverse effects on liver enzymes or other cardiometabolic markers [27]. Clinical safety has been addressed directly in a single dedicated study (*n* = 14 completers, seven female/seven male, from 16 enrolled; within-subject design, each participant serving as their own control, no separate placebo arm): six days of *S. scardica* decoction, probed with caffeine and paracetamol, left CYP1A2, xanthine oxidase, N-acetyltransferase-2, and UGT1A1/1A6 activities unchanged, indicating minimal herb–drug-interaction risk for most common pharmaceuticals, whereas a statistically significant reduction in CYP2A6 activity (*p* < 0.05) occurred in male subjects only [31]. Results are reported as enzyme-activity comparisons with significance testing; confidence intervals and effect sizes were not provided [31]. This remains the only published clinical safety evaluation of any *Sideritis* species and, together with the sex-stratified insulin effect noted in Section 4.2, argues for sex-disaggregated analysis in future work.

Pharmacokinetic data are similarly sparse. Urinary excretion of *Sideritis* polyphenols after mountain-tea consumption has been characterized in the only published absorption study [32], but bioavailability from different delivery matrices has not been compared directly. The importance of this gap is illustrated by a randomized, double-blind, placebo-controlled trial (*n* = 51 completers, from 63 recruited; 30-day intervention) in which a low-dose (0.885 mg total flavonoids) *S. euboea* preparation produced no significant between-group change in any measured antioxidant biomarker in healthy adults [33]—a null result demonstrating that in vitro antioxidant activity does not automatically translate to in vivo biomarker change, and leaving the effective dose of *Sideritis* polyphenols unresolved [33]. Closing this gap will require pharmacokinetic work that moves beyond urinary recovery: simulated gastrointestinal digestion to model the stability and release of verbascoside and the major flavonoids, and intestinal-permeability assays (e.g., Caco-2 transport models) to establish whether these constituents reach their proposed molecular targets at physiologically relevant concentrations. Conversely, co-delivery with fructo-oligosaccharides produced significant bifidogenic effects on fecal microbiota—the first in vivo evidence that *Sideritis* polyphenols may act partly through the gut microbiome, a route increasingly recognized as mediating polyphenol-related health effects [34].

Two further gaps are critical for translation. First, beyond the herb–drug-interaction screening above, systematic acute, sub-chronic, and chronic toxicology is essentially absent: no genotoxicity, reproductive, or developmental studies have been reported for any *Sideritis* species, and pediatric and pregnancy data are entirely lacking. Second, chemical heterogeneity driven by genotype, altitude, harvest timing, drying, and storage represents a fundamental standardization challenge; verbascoside, isoscutellarein 7-O-allosyl-(1→2)-glucoside, chlorogenic acid, and kaempferol are the most frequently proposed marker compounds, but no harmonized pharmacopeial standard has been adopted. Resolving both is a prerequisite for reproducible mechanistic studies and for any future therapeutic development of the genus. A practical priority is comparative, LC-MS/MS-based metabolomic fingerprinting of the major *Sideritis* species, analyzed together with their biological activity, so that metabolite abundance can be correlated with bioactivity to identify genuine marker compounds and anchor standardized preparations.

**Figure 4:**
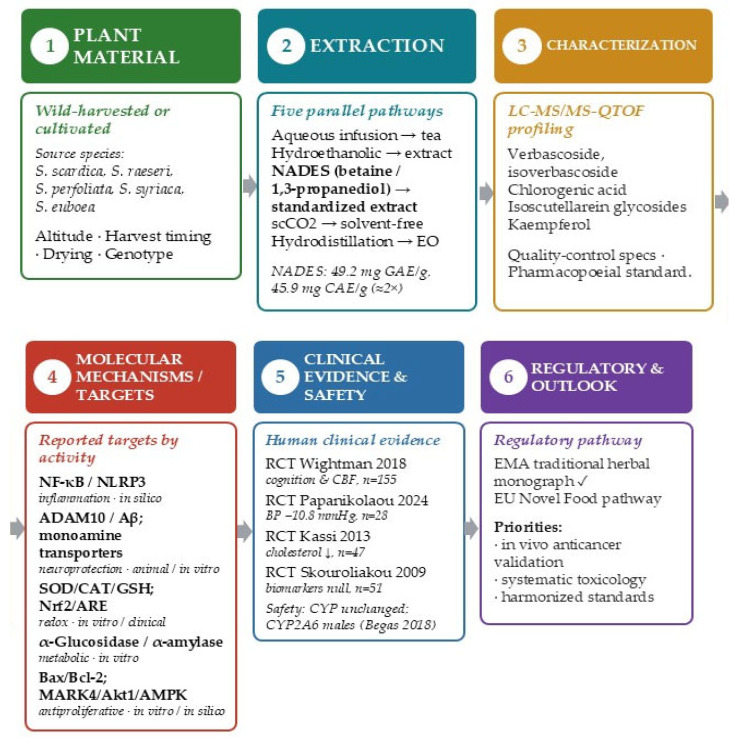
Translation of *Sideritis* bioactive compounds from characterization to potential therapeutic application. ↓—decrease [31].

The translation of *Sideritis* species from traditional ethnobotanical use to standardized food and nutraceutical products represents one of the most promising and least systematically explored aspects of this genus (Figure 4).

## 6. Conclusions

*Sideritis* species, and *S. scardica* in particular, are emerging as a coherent group of plants whose long-standing reputation as a remedy is now resolving into defined molecular events. Their phenylethanoid glycosides, flavonoids, and phenolic acids act across redox, inflammatory, neuroprotective, cardiometabolic, and antiproliferative pathways, with verbascoside the single best-characterized constituent—the only one shown by direct in vitro binding (nanomolar-to-micromolar affinity for NF-κB and NLRP3) to engage the checkpoints that link anti-inflammatory and cancer-relevant activity. Neuroprotection (amyloid-β processing via ADAM10 and triple monoamine-transporter inhibition), carbohydrate-enzyme inhibition, and apoptotic/kinase targets (Bax, Akt1, AMPK, MARK4) complete a multi-target profile, the antiproliferative arm of which remains, for now, only at the cell-based and computational level.

What distinguishes the genus from many traditionally used botanicals is the convergence of in silico prediction, in vitro and direct-binding data, in vivo disease models, and small randomized clinical trials of standardized *S. scardica* reporting cognitive, anxiolytic, cardiometabolic, and redox benefits, together with clinical evidence of minimal herb–drug-interaction risk. This makes *Sideritis* a promising, mechanistically grounded source of bioactive compounds for inflammatory, neurodegenerative, and metabolic disorders—and a credible candidate for standardized food and nutraceutical development.

Realizing this potential depends on closing three gaps: in vivo validation of the antiproliferative and anticancer effects before any therapeutic claim; systematic toxicology, including genotoxicity, reproductive, and developmental endpoints with sex-stratified analysis; and phytochemical standardization around consensus marker compounds (verbascoside, isoscutellarein glycosides, chlorogenic acid, kaempferol). Addressing these priorities would consolidate the genus as a well-defined contributor to the study of bioactive compounds in human health and disease.

## Figures and Tables

**Table 1:** Major bioactive compounds identified in *Sideritis* species: compound class, key structural features, quantitative concentration data (where available), species of occurrence, analytical method, and primary references.

Compound	Compound Class	Key Structural Feature	Concentration Range	Species Detected	Analytical Method	Key References
Verbascoside (acteoside)	Phenylethanoid glycoside	Caffeoyl ester; catechol ring; rhamnosyl-glucoside	† 136.8–50,951.1 µg/g (water >> MeOH >> EtOAc)	*S. perfoliata*, *S. scardica*, *S. raeseri*, *S. syriaca*, *S. cypria*	LC-ESI-MS/MS; LC-MS/MS-QTOF; NMR	[5,6,10]
Chlorogenic acid	Phenolic acid (hydroxycinnamic)	Ester of caffeic acid and quinic acid	† 11.0–24,933.4 µg/g (water >> MeOH >> EtOAc)	*S. perfoliata*, *S. scardica*, *S. raeseri*, *S. syriaca*, *S. cypria*	LC-ESI-MS/MS; LC-MS/MS-QTOF; NMR	[5,6,10]
Apigenin 7-O-glucoside	Flavone glycoside	Glucosylation at C-7; enhanced solubility	† 278.4–1437.5 µg/g; det. in all 9 populations	*S. perfoliata*, *S. scardica*, *S. raeseri*, *S. syriaca*, *S. pisidica*	LC-ESI-MS/MS; LC-MS/MS-QTOF	[5,10]
Isoscutellarein 7-O-[6″-O-acetyl]-allosyl(1 → 2)-glucoside	Flavone diglycoside	Rare allosyl-glucoside; acetylation; genus-characteristic	det. in all 9 populations (no conc. reported)	*S. scardica*, *S. raeseri*, *S. syriaca*	LC-MS/MS-QTOF	[5]
Apigenin 7-(4″-p-coumaroylglucoside)	Acylated flavone glycoside	Coumaroyl acylation; delayed enzymatic hydrolysis	det. in all 9 populations (no conc. reported)	*S. scardica*, *S. raeseri*, *S. syriaca*	LC-MS/MS-QTOF	[5]
Apigenin	Flavone (aglycone)	4′,5,7-trihydroxyflavone; three OH groups	† 1.5–35.4 µg/g; det. in 7/9 populations	*S. perfoliata*, *S. scardica*, *S. raeseri*, *S. syriaca*, *S. pisidica*	LC-ESI-MS/MS; LC-MS/MS-QTOF	[5,10]
Luteolin	Flavone (aglycone)	3′,4′,5,7-tetrahydroxyflavone; catechol B-ring	n.q.	*S. scardica*, *S. cypria*, *S. pisidica*	NMR; LC-MS	[6,9]
Luteolin 7-glucoside	Flavone glycoside	C-7 glucosylation of luteolin	† 2.45–6.23 µg/g	*S. perfoliata*, *S. pisidica*	LC-ESI-MS/MS	[10]
Kaempferol	Flavonol	3-hydroxyflavone; C-3 hydroxyl group	n.q.	*S. pisidica*	LC-MS	[9]
Hesperidin	Flavanone glycoside	Rutinoside disaccharide; methoxylated B-ring	† 0.82 µg/g (MeOH only)	*S. perfoliata*, *S. pisidica*	LC-ESI-MS/MS	[10]
Hyperoside	Flavonol glycoside	Quercetin 3-O-galactoside	† 2.20–5.12 µg/g	*S. perfoliata*, *S. pisidica*	LC-ESI-MS/MS	[10]
Rosmarinic acid	Phenolic acid (hydroxycinnamic)	Two catechol moieties; four phenolic OH groups	† 1.52 µg/g (EtOAc only; absent in water/MeOH)	*S. perfoliata*, *S. pisidica*, *S. scardica*	LC-ESI-MS/MS; LC-MS	[9,10]
Caffeic acid	Phenolic acid (hydroxycinnamic)	Single catechol; two phenolic OH groups	† 0.53–186.8 µg/g	*S. perfoliata*, *S. pisidica*	LC-ESI-MS/MS	[10]
Lavandulifolioside	Phenylethanoid glycoside	Trisaccharide chain; arabinose unit	det. (leaves only)	*S. cypria*, *S. raeseri* (SR2 only)	NMR; LC-MS/MS-QTOF	[5,6]
Leucosceptoside A	Phenylethanoid glycoside	Methoxylated caffeoyl group	det.	*S. cypria*	NMR	[6]
Leonoside A	Phenylethanoid glycoside	Trisaccharide with arabinose	det.	*S. cypria*	NMR	[6]
Lamalboside	Phenylethanoid glycoside	Trisaccharide with galactose (rare in genus)	det.	*S. cypria*	NMR	[6]
Melittoside	Iridoid glycoside	Cyclopentane-fused pyran; glucoside	det.	*S. cypria*	NMR	[6]
Pinoresinol	Lignan	Furofuran-type; two guaiacyl units	† 4.96–12.18 µg/g	*S. perfoliata*	LC-ESI-MS/MS	[10]
α-Pinene	Monoterpene hydrocarbon	Bicyclic; most abundant monoterpene in genus	4.3–38.0% (EO); flower > leaf in *S. cypria*	*S. cypria*, *S. scardica*, *S. raeseri*, *S. perfoliata*, +many spp.	GC-MS	[6,7]
β-Phellandrene	Monoterpene hydrocarbon	Monocyclic; ∼25% in *S. cypria* (both organs)	17.8–25.8% (EO)	*S. cypria*, *S. perfoliata*	GC-MS	[6,7]
β-Caryophyllene	Sesquiterpene hydrocarbon	Bicyclic; selective CB2 agonist; FDA food additive	2.6–22.5% (EO); leaf >> flower in *S. cypria*	*S. cypria*, *S. scardica*, *S. raeseri*, *S. perfoliata*, +many spp.	GC-MS	[6,7]
Carvacrol	Monoterpenoid phenol	Phenolic OH on cymene ring	up to 33.7% (EO) in *S. syriaca* subsp. syriaca	*S. syriaca*, *S. condensata*, *S. raeseri (Albania)*	GC-MS	[7]
Caryophyllene oxide	Oxygenated sesquiterpene	Epoxide of β-caryophyllene	1.5–19.6% (EO)	*S. cypria*, *S. arborescens*, *S. scardica*, +many spp.	GC-MS	[6,7]

Concentrations marked † are from Sarikurkcu et al. (2020) on *S. perfoliata* (LC-ESI-MS/MS, µg/g dry extract, range across ethyl acetate/methanol/water) [10]. Entries marked “det.” indicate qualitative detection only. Note on apigenin 7-(4″-p-coumaroylglucoside): detected in all nine populations across *S. raeseri*, *S. scardica*, and *S. syriaca* by Kaparakou et al. (2024), but no quantitative concentration has been reported in any published study—a specific research gap, since acylated flavone glycosides may exhibit enhanced stability and bioavailability, making their quantification important for mechanistic and pharmacological standardization [5].

**Table 2:** Major biological activities of *Sideritis* species and their proposed mechanisms of action, with key quantitative evidence.

Biological Activity	Species	Preparation/ Compound	Experimental Model	Dose/ Concentration	Comparator	Key Quantitative Result	Evidence Type	Ref
Antioxidant	*S. perfoliata*	Water extract (verbascoside/phenolic-rich)	In vitro (DPPH, ABTS, CUPRAC, FRAP)	Extract	Trolox	DPPH 405.53; ABTS 149.71; CUPRAC 210.69; FRAP 161.19 mg TE/g	In vitro	[10]
Antioxidant	*S. scardica*	Raw material, 4 origins	In vitro (DPPH)	-	-	86.5–87.9% DPPH inhibition; verbascoside predominant	In vitro	[17]
Antioxidant	*S. scardica*	SidTea+™ aqueous extract	Human RCT (*n* = 28)	1500 mg/day, 4 wk	Placebo	↑ total antioxidant capacity (*p* < 0.001); ↓ plasma lipid peroxidation (*p* < 0.05)	Clinical	[18]
Anti-inflammatory	*S. scardica*	Diethyl ether & n-butanol fractions	In vivo (carrageenan paw edema, rat)	100–200 mg/kg	Indomethacin (4 mg/kg)	~50% edema reduction; comparable to indomethacin	Animal	[18]
Anti-inflammatory	*S. bilgeriana*	Methanolic extract	In vivo (pleurisy; neuropathic pain)	-	-	↓ TNF-α, IL-1β; NF-κB suppression; ↓ cell migration	Animal	[19]
Anti-inflammatory	Genus (verbascoside)	Isolated verbascoside	In silico screen + in vitro MST	-	-	Top dual binder of NLRP3 and NF-κB among 657 screened metabolites (in silico)	In silico	[20]
Antimicrobial	*S. syriaca* subsp. syriaca	Essential oil (carvacrol 33.7%)	In vitro (MIC, 6 bacteria)	0.60–2.25 mg/mL	Carvacrol/pinene alone	Strongest antibacterial oil in the review, MIC 0.60–2.25 mg/mL across six Gram-positive/Gram-negative species; exceeds carvacrol or α-/β-pinene alone (synergy)	In vitro	[7]
Antimicrobial	*S. bilgeriana*	Essential oil (β-pinene-rich)	In vitro (MIC, *C. albicans*)	0.03 mg/mL	Ketoconazole	Pinene-rich chemotype: anti-Candida albicans MIC 0.03 mg/mL, matching ketoconazole	In vitro	[7]
Antimicrobial	*S. scardica*	Polar extracts (multi-origin)	In vitro	-	-	Activity vs. Gram-positive & Candida; Türkiye origin strongest	In vitro	[17]
Neuroprotective	*S. scardica*, *S. euboea*	Oral extracts (individual + combined)	In vivo (APP-transgenic mice)	Daily, post-onset	Vehicle	Aβ42 ↓ 56–60%; plaques ↓ 42%; escape latency ↓ 55–61%; ADAM10 ↑, microglia ↑	Animal	[21]
Neuroprotective	*S. scardica*	Methanol extract	In vitro (synaptosomes; hSERT JAR cells)	EC50 31–42 µg/mL; 1.4 µg/mL (hSERT)	Fluvoxamine	Triple monoamine reuptake inhibition; SSRI synergy	In vitro	[22]
Neuroprotective/cognitive	*S. scardica*	Standardized extract	Human RCT (*n* = 155)	475/950 mg/day, 28 d	Ginkgo biloba 240 mg; placebo	↓ RVIP false alarms; ↓ state anxiety; ↑ prefrontal HbO (*p* < 0.05)	Clinical	[23]
Hepatoprotective	*S. congesta*	Extract	In vitro (APAP-treated HepG2)	-	-	Restored SOD/CAT; maintained GSH; ↓ MDA; anti-mutagenic	In vitro	[12]
Hepatoprotective	*S. scardica*	SidTea+™ aqueous extract	Human RCT (*n* = 28)	1500 mg/day, 4 wk	Placebo	↓ γ-GT; ↓ SGPT (*p* < 0.05)	Clinical	[24]
Gastroprotective	*S. scardica*	n-butanol & diethyl ether extracts	In vivo (ethanol-induced gastric damage, rat)	Dose-dependent	Ranitidine	Dose-dependent protection comparable to ranitidine	Animal	[18]
Gastroprotective	*S. perfoliata*	Ethanolic extract	In vivo (acetic-acid colitis, rat)	200 mg/kg	Sulfasalazine	↓ TNF-α, IL-1β, IL-17; ↓ TLR-9, MMP-3, caspase-3/9	Animal	[25]

Evidence type: in vitro = cell-free/cell-based assay; Animal = in vivo model; Clinical = human trial; In silico = computational prediction. ↑—increase; ↓—decrease.

## Data Availability

No new data were created or analyzed in this study. Data sharing is not applicable to this article.

## Abbreviations

ABTS	2,2′-azino-bis(3-ethylbenzothiazoline-6-sulfonic acid) assay
AChE	acetylcholinesterase
ADAM10a	disintegrin and metalloproteinase domain-containing protein 10 (α-secretase)
ADHD	attention-deficit/hyperactivity disorder
Akt RAC	alpha serine/threonine protein kinase (protein kinase B α)
AMPK	AMP-activated protein kinase
APAF	apoptotic protease activating factor
APAP	acetaminophen
APP	amyloid precursor protein
ARE	antioxidant response element
Aβ/Aβ42	amyloid-β/amyloid-β (1–42) peptide
Bax	Bcl-2-associated X protein
Bcl-2	B-cell lymphoma 2
BChE	butyrylcholinesterase
BP	blood pressure
CAE	caffeic acid equivalents
CAT	catalase
CB2	cannabinoid receptor type 2
CBF	cerebral blood flow
CI	confidence interval
CUPRAC	cupric reducing antioxidant capacity
CYP1A2/CYP2A6	cytochrome P450 1A2/2A6
DEF-β2	β-defensin 2 (human beta-defensin 2)
DNA	deoxyribonucleic acid
DPPH	2,2-diphenyl-1-picrylhydrazyl
DW	dry weight
EC_50_	half-maximal effective concentration
EMA	European Medicines Agency
EO	essential oil
EtO	Acethyl acetate
FDA(U.S.)	Food and Drug Administration
FRAP	ferric reducing antioxidant power
GAE	gallic acid equivalents
GC-MS	gas chromatography–mass spectrometry
GRAS	generally recognized as safe
GSH	reduced glutathione
HbO	oxygenated hemoglobin
HOMA-IR	homeostatic model assessment of insulin resistance
HPLC	high-performance liquid chromatography
hSERT	human serotonin transporter
IC_50_	half-maximal inhibitory concentration
IL-1β/IL-17	interleukin-1 beta/interleukin-17
Kd	(K_d) dissociation constant
LC-ESI-MS/MS	liquid chromatography–electrospray ionization–tandem mass spectrometry
LC-MS/MS(-QTOF)	liquid chromatography–tandem mass spectrometry (–quadrupole time-of-flight)
MARK4 MAP	microtubule affinity-regulating kinase 4
MDA	malondialdehyde
MeOH	methanol
MIC	minimum inhibitory concentration
MMP-3	matrix metalloproteinase-3
MST	microscale thermophoresis
NADES	natural deep eutectic solvent
NCI	National Cancer Institute
NF-κB	nuclear factor kappa-light-chain-enhancer of activated B cells
NLRP3	NLR family pyrin domain-containing protein 3 (inflammasome)
NMR	nuclear magnetic resonance
Nrf2	nuclear factor erythroid 2–related factor 2
QE	quercetin equivalents
RCT	randomized controlled trial
ROS	reactive oxygen species
RVIP	Rapid Visual Information Processing
scCO_2_	supercritical carbon dioxide
SEM	standard error of the mean
SGPT	serum glutamic-pyruvic transaminase (alanine aminotransferase)
SOD	superoxide dismutase
SSRI	selective serotonin reuptake inhibitor
TBARS	thiobarbituric acid reactive substances
TE	Trolox equivalents
TGF-β	transforming growth factor beta
TLR-9	Toll-like receptor 9
TNF-α	tumor necrosis factor alpha
UGT1A1/1A6	UDP-glucuronosyltransferase 1A1/1A6
UPLC-HRMS	ultra-(high-)performance liquid chromatography–high-resolution mass spectrometry
UV	ultraviolet
VO_2_max	maximal oxygen uptake
γ-GT	gamma-glutamyl transferase
5-LOX	5-lipoxygenase
